# Sub-micrometre focusing of intense 100 keV X-rays with multilayer reflective optics

**DOI:** 10.1107/S1600577524000213

**Published:** 2024-02-22

**Authors:** Takahisa Koyama, Hirokatsu Yumoto, Takanori Miura, Yasuhisa Matsuzaki, Makina Yabashi, Haruhiko Ohashi

**Affiliations:** a Japan Synchrotron Radiation Research Institute (JASRI), 1-1-1 Kouto, Sayo, Hyogo 679-5198, Japan; b RIKEN SPring-8 Center, 1-1-1 Kouto, Sayo, Hyogo 679-5148, Japan; Bhabha Atomic Research Centre, India

**Keywords:** high-energy X-rays, 100 keV X-rays, multilayer monochromators, multilayer focusing mirrors, scanning transmission imaging

## Abstract

A high-flux sub-micrometre focusing system was constructed using multilayer focusing mirrors in Kirkpatrick–Baez geometry for 100 keV X-rays. The focusing mirror system had a wide bandwidth of 5% and a high peak reflectivity of 74%. Performance was evaluated at the undulator beamline BL05XU of SPring-8, which produced an intense 100 keV X-ray beam with a bandwidth of 1%. When the light source was focused directly in both vertical and horizontal directions, the beam size was measured to be 0.32 µm (V) × 5.3 µm (H) with a flux of 1 × 10^12^ photons s^−1^. However, when a limited horizontal slit was used to form a secondary source, the focusing beam size decreased to 0.25 µm (V) × 0.26 µm (H) with a flux of 6 × 10^10^ photons s^−1^. The 200 nm line and space patterns of a Siemens star chart made of tantalum were clearly resolved by the absorption contrast of the focused beam. This 100 keV focusing system is applicable to various fields of nondestructive analyses with sub-micrometre resolutions.

## Abstract 2

A high-flux sub-micrometre focusing system for 100 keV X-rays using multilayer focusing mirrors was constructed and evaluated at the undulator beamline BL05XU of SPring-8.

## 1. Introduction

High-energy X-ray beams are powerful tools for nondestructively analyzing thick heavy metals, devices enclosed within exterior cases and materials in high-pressure cells. For high-energy X-ray applications such as transmission X-ray imaging, X-ray fluorescence spectroscopy and total-scattering measurements with pair distribution function analysis, a high energy resolution of the incident X-ray beam below 0.1% is not necessary. Instead, a ‘pink’ beam with a modest resolution of 1–10% is preferable because it enhances the photon flux. The focus of such a high-flux beam in a small region enables high spatiotemporal resolution analysis.

Several types of devices have been developed for focusing high-energy X-rays above 50 keV, such as Fresnel zone plates (FZPs) (Kamijo *et al.*, 2003 ▸; Snigireva *et al.*, 2007 ▸; Tamura *et al.*, 2009 ▸), multilayer Laue lenses (MLLs) (Li *et al.*, 2023 ▸), compound refractive lenses (CRLs) (Snigirev *et al.*, 2004 ▸, 2007 ▸; Vaughan *et al.*, 2010 ▸; Shastri *et al.*, 2014 ▸, 2020 ▸; Brancewicz *et al.*, 2016 ▸; Hirao & Ohishi, 2022 ▸) and total-reflection Kirkpatrick–Baez (KB) optics (Suzuki *et al.*, 2007 ▸; Hayashi *et al.*, 2016 ▸). Both FZPs and CRLs are on-axis devices keeping simple optical geometry, while they have chromatic aberration with a reduced throughput. MLLs have a high diffraction efficiency with a small focus size, although they also have chromatic aberration with a small acceptance aperture. The chromatic aberrations of these devices are proportional to the photon energy *E* (*E* ^2^) for FZPs and MLLs (CRLs), limiting generation of a small focus with an intense pink beam due to a restriction of the numerical aperture (NA). Total-reflection KB optics have achromatic properties but the critical angle becomes extremely small for high-energy X-rays, resulting in small spatial acceptance and low throughput. Finally, multilayer KB optics can be designed to accept a reasonably wide bandwidth of a few percent at a specific photon energy, which can provide an intense small focus with a large spatial acceptance, large NA and high reflectivity. However, fabrication of such focusing devices for high-energy X-rays of ∼100 keV is still challenging because it requires precise fabrication of surface profiles and precise control of multilayer deposition.

So far, there are a few reports on focusing high-energy (above 50 keV) X-rays using multilayer focusing mirrors. One-dimensional focusing of the 10 µm range with 65.4 and 76.6 keV X-rays has been reported by using a multilayer coated bimorph mirror (Sutter *et al.*, 2019 ▸). Two-dimensional sub-micrometre focusing with 20–69 keV X-rays has been realized by using multilayer coated dynamically figured mirrors (Vaughan *et al.*, 2020 ▸). Furthermore, 67.7 keV X-rays have been planned to focus down to the sub-micrometre range (Archilha *et al.*, 2022 ▸).

In this study, we developed a 100 keV KB focusing system composed of laterally graded multilayers on high-precision figured mirrors. The focusing mirror system had a wide bandwidth of 5% and a high peak reflectivity of 74%. Performance was evaluated at the undulator beamline BL05XU of SPring-8, which produced an intense 100 keV X-ray beam with a bandwidth of 1%. The design, fabrication and evaluation results are presented in this article.

## 2. Design of a high-flux 100 keV focusing system

**Figure 1:**
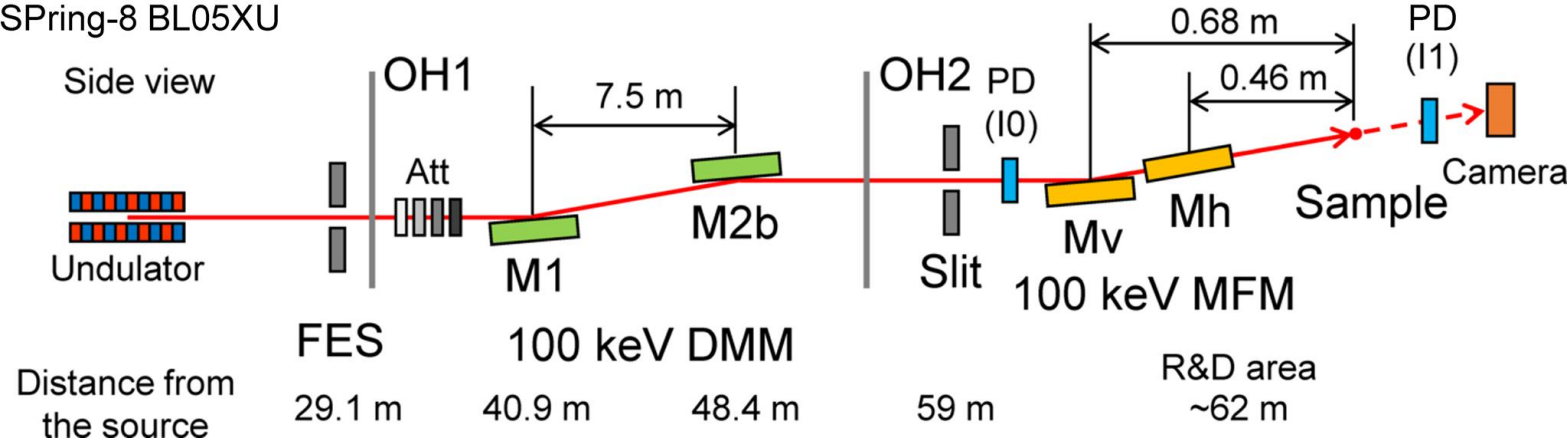
Layout of the main optical components of optics hutches (OH1 and OH2) at the SPring-8 BL05XU beamline. The label references are as follows: FES = frontend slit, Att = attenuators, DMM = double multilayer monochromator, MFM = multilayer focusing mirror and PD = photodiode detector.

**Figure 2:**
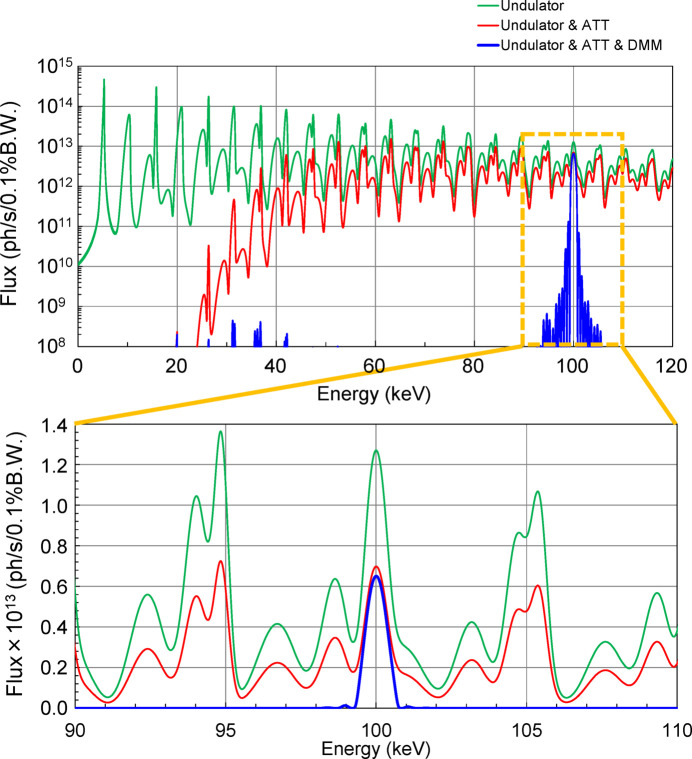
The calculated undulator spectrum (green line), that with attenuators (red line), and that with attenuators and DMM (blue line). A log-scale graph with a 0–120 keV energy range (top). An enlarged graph of the top in linear scale with a 90–110 keV energy range (bottom).

Fig. 1 ▸ shows the layout of the main optical components in the optics hutches (OH1 and OH2) of the BL05XU beamline (Yumoto *et al.*, 2020 ▸), which has a SPring-8 standard in-vacuum undulator with a period length of 32 mm. In this study, the 19th harmonic of the 5.3 keV fundamental radiation, which has an energy width of 0.93%, was used. The spatial profile at the source point was calculated to be 15.2 µm (V) × 747 µm (H) in full width at half-maximum (FWHM) at 100 keV with 1% bandwidth using the *SPECTRA* software (Tanaka & Kitamura, 2001 ▸). The entire spectrum of the 19th harmonic was extracted using a double multilayer monochromator (DMM). The total-reflection low energy (<30 keV) component after the DMM was suppressed by attenuators (Yumoto *et al.*, 2020 ▸). The calculated spectra of the undulator source after the frontend slit (FES), attenuators and the DMM are shown in Fig. 2 ▸. A clean single-peak spectrum was obtained. A photon flux of 3 × 10^13^ photons s^−1^ with an energy bandwidth of 1% was achieved at 100 keV (Yumoto, 2024 ▸). The power after the DMM was suppressed to be 0.5 W.

**Figure 3:**
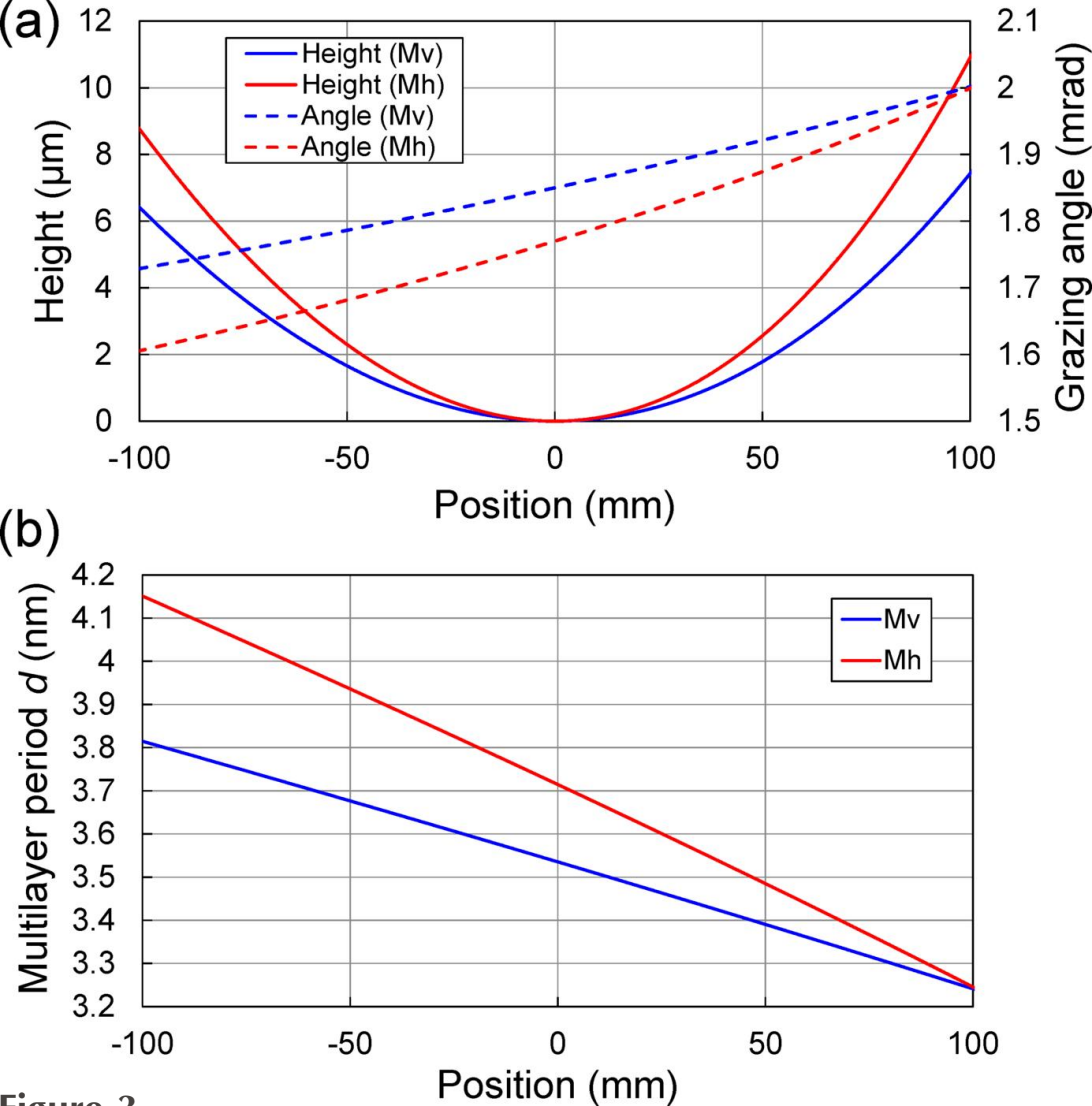
(*a*) Height profiles and grazing-angle distributions and (*b*) multilayer period distributions along the position of the focusing mirrors.

**Figure 4:**
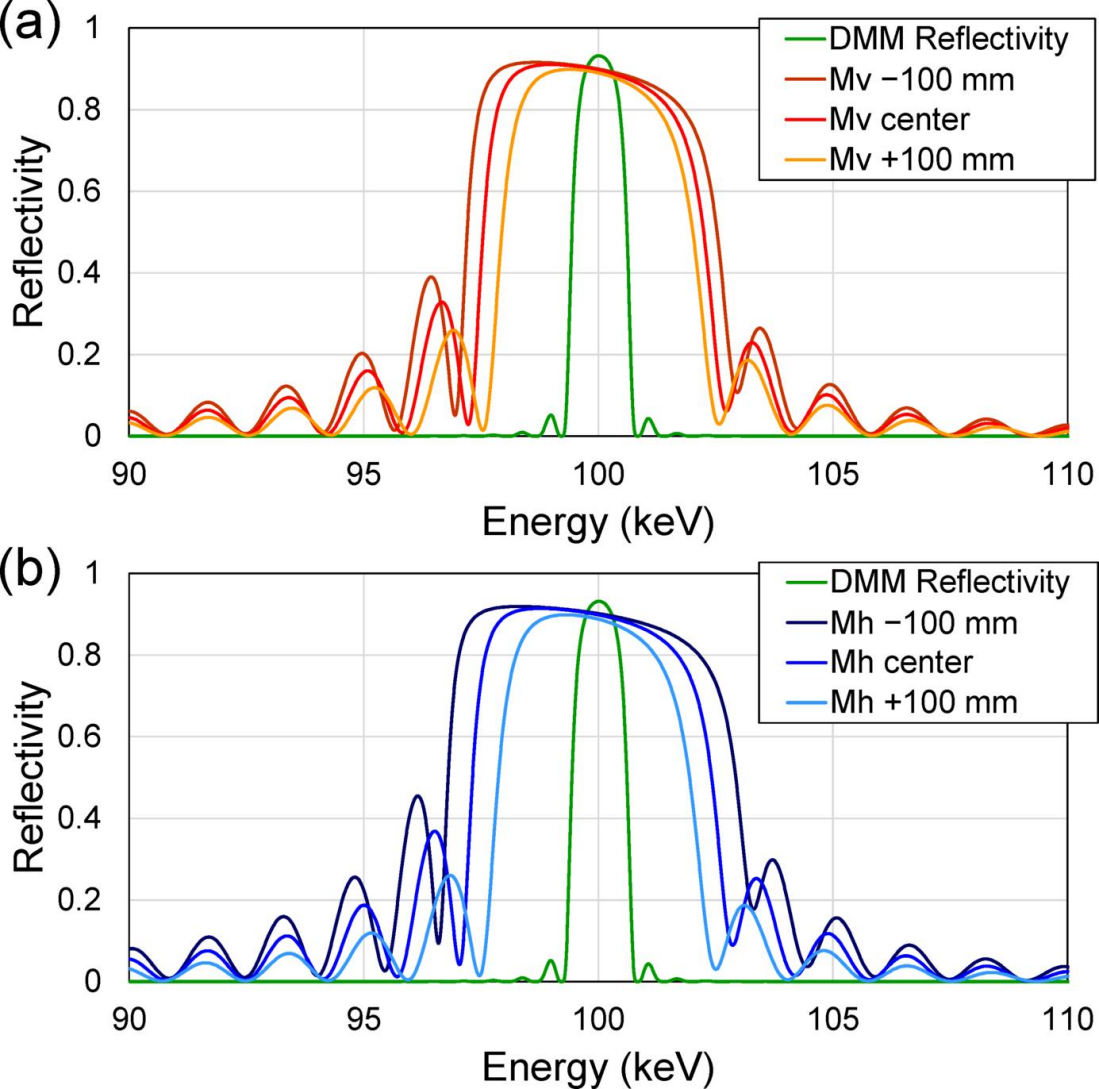
Reflectivity curves of the DMM (double reflection), (*a*) vertical (Mv) and (*b*) horizontal (Mh) focusing mirrors. Three curves of upstream edge (−100 mm), center and downstream edge (+100 mm) parts of (*a*) Mv and (*b*) Mh are plotted. The energy range of 90–110 keV is shown.

We designed laterally graded multilayer focusing mirrors with a [W/C]_50_ coating to generate a sub-micrometre beam size in the vertical direction. A similar size was achieved in the horizontal direction when the horizontal size of the FES was restricted as a secondary source. The parameters of the focusing mirrors are listed in Table 1 ▸. The thickness ratio was chosen to be 0.5. The first reason for this is that the reflectivity change at an X-ray energy above 100 keV is small around the thickness ratio of 0.5, because the effect of the absorption of high-*Z* material is small. The second reason is to reduce second-order reflection. The height profiles, grazing-angle distributions and multilayer period distributions of the focusing mirrors are shown in Fig. 3 ▸. From the source size and the demagnification factor, the geometric focusing size is calculated to be 0.17 µm (V) × 5.5 µm (H) in FWHM when the light source is directly focused, while 0.28 µm (H) when the horizontal size of the FES is closed to be 20 µm. The calculated reflectivity curves of the DMM, vertical focusing mirror (Mv) and horizontal focusing mirror (Mh) are shown in Fig. 4 ▸. The bandwidth along the entire length of the multilayer focusing mirrors (∼5%) was designed to cover the full bandwidth of the DMM (1%) with sufficient tolerance for possible alignment errors and/or multilayer deposition errors.

## 3. Results

### 3.1. Deposition results

The substrate surfaces of the mirrors were finished by the JTEC Corporation and the multilayer coatings were deposited at our SPring-8 in-house laboratory. The slope error of each mirror substrate was evaluated to be 0.060 (0.025) µrad in RMS for Mv (Mh) by stitching interferometry over the full length of the effective area and low-pass filtering at a spatial wavelength of 20 mm, which is equivalent to one-tenth of the mirror length of 200 mm. The slope error had a significant effect on the focusing size, and the blurring of the focusing size due to the slope error was estimated to be 0.19 µm (V) × 0.056 µm (H) in FWHM. This is sufficiently smaller than sub-micrometre focusing size. Surface-roughness values were determined to be 0.2 nm in RMS (evaluation area: 170 µm × 100 µm) using a white light interferometer. Although this value affects the roughness of the multilayer, its effect on reflectivity is insignificant.

The deposition system is based on direct current magnetron sputtering and was designed to coat up to 600 mm long and 50 mm wide substrates. The system consists of a main chamber for the coating process and a load lock chamber for sample loading without changing the main-chamber environment. The main chamber contains four magnetron-sputtering cathodes with 2-inch targets. The substrates can be moved in front of the sputter sources in order to realize long coatings and thickness gradients. Furthermore, 50 mm square slits with an arc-shaped mask attached were used to limit sputter particles inside the mirror surface and to improve thickness distribution in the sagittal direction. The thickness control along the substrate motion direction is based on speed variation as it moves in front of the sputter source, while all other deposition parameters are kept constant (Morawe & Peffen, 2009 ▸). The period length of the multilayer was varied from 3.24 to 3.8 nm for Mv and 3.24 to 4.15 nm for Mh, as shown in Fig. 3 ▸(*b*). The discharge powers were 50 W for tungsten and 60 W for carbon. To increase the deposition rate of carbon, two sputter cathodes were used to deposit concurrently on one deposition area. The process gas was argon with a vacuum of 0.1 Pa. A chromium layer was inserted between the substrate and the multilayer to improve adhesion and allow recoating to be performed.

**Figure 5:**
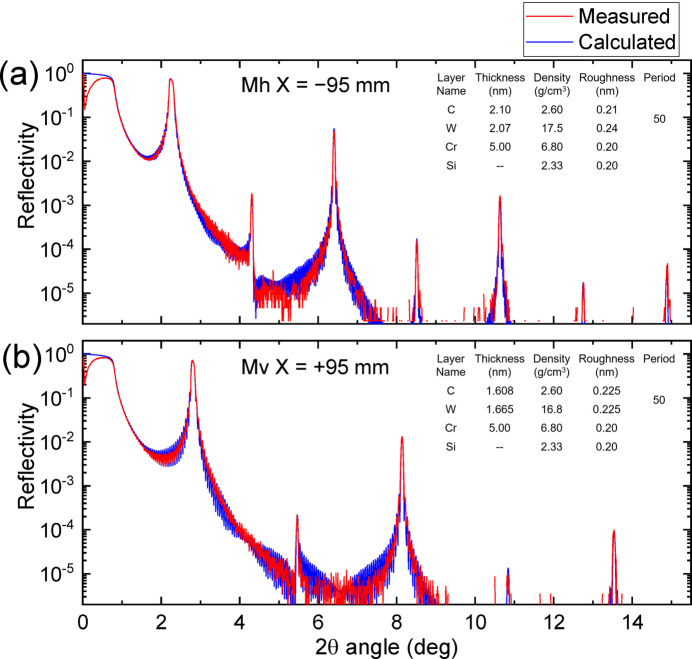
The measured and calculated reflectivity curves plotted as a function of 2θ angle at (*a*) a position of −95 mm on Mh and (*b*) a position of +95 mm on Mv for *E* = 8048 eV. The parameters used in the calculation are shown in the graphs.

**Figure 6:**
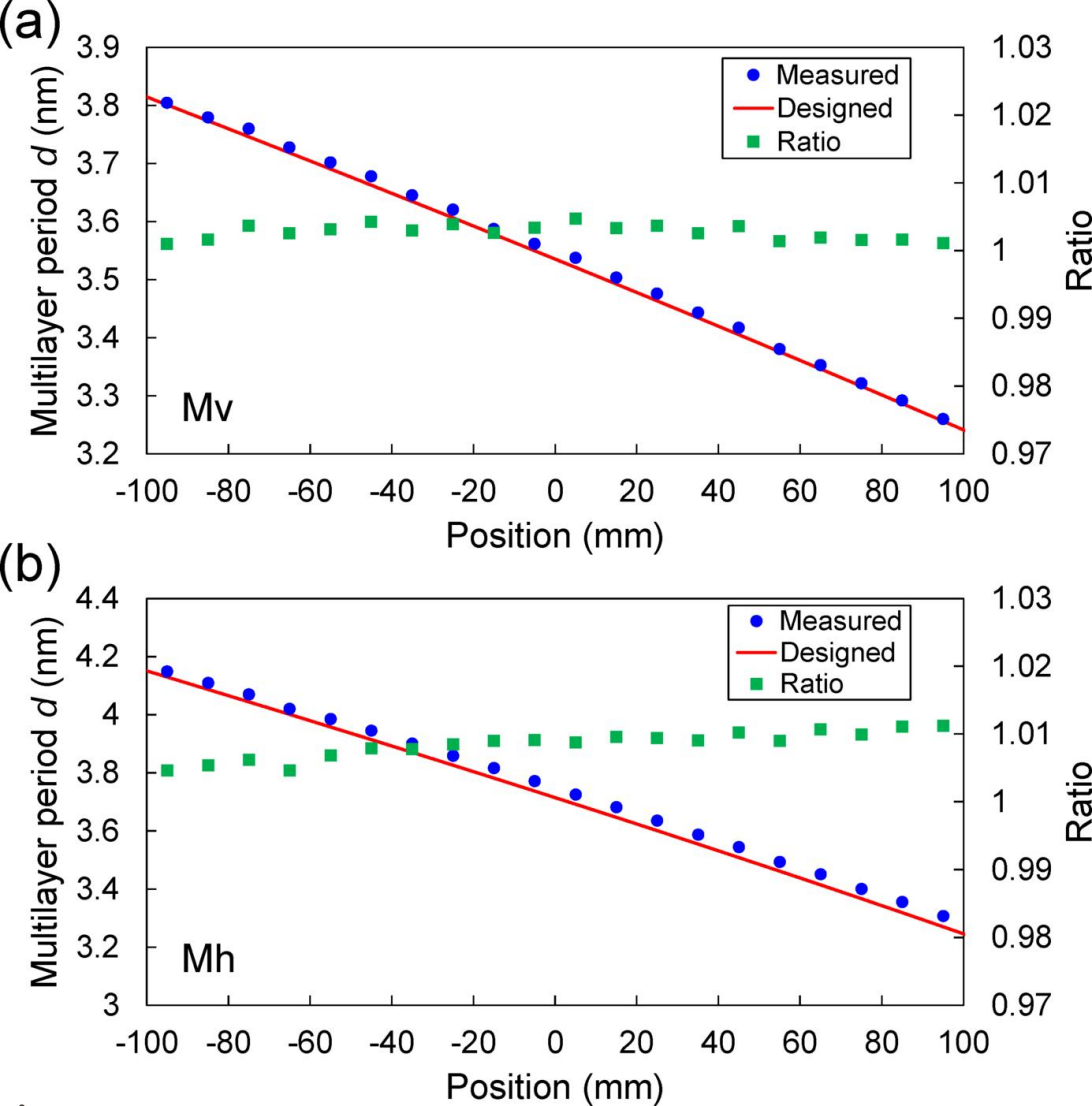
The distribution of multilayer periods *d* evaluated at each position on (*a*) Mv and (*b*) Mh.

The deposition results of the multilayers are shown in Figs. 5 ▸ and 6 ▸, and were evaluated by X-ray reflectivity using a Cu *K*α source for Si test-piece wafers located beside the high-precision figured area of the substrate. The measured and calculated reflectivity curves were plotted as a function of 2θ angle at the position of −95 mm on Mh and the position of +95 mm on Mv, as shown in Fig. 5 ▸. The measured curves were almost reproduced by the calculated curves using the parameters shown in the graph. The multilayer periods *d* evaluated at each position on Mv and Mh are shown in Fig. 6 ▸. The error in the multilayer period was almost within ±1%. This error was tolerated because the reflection energy width of the multilayer was as broad as ∼5%, as shown in Fig. 4 ▸. Measured interface-roughness values of the multilayer were ∼0.21–0.25 nm in RMS, which has a small effect on reflectivity.

### 3.2. Focusing-beam characterization

**Figure 7:**
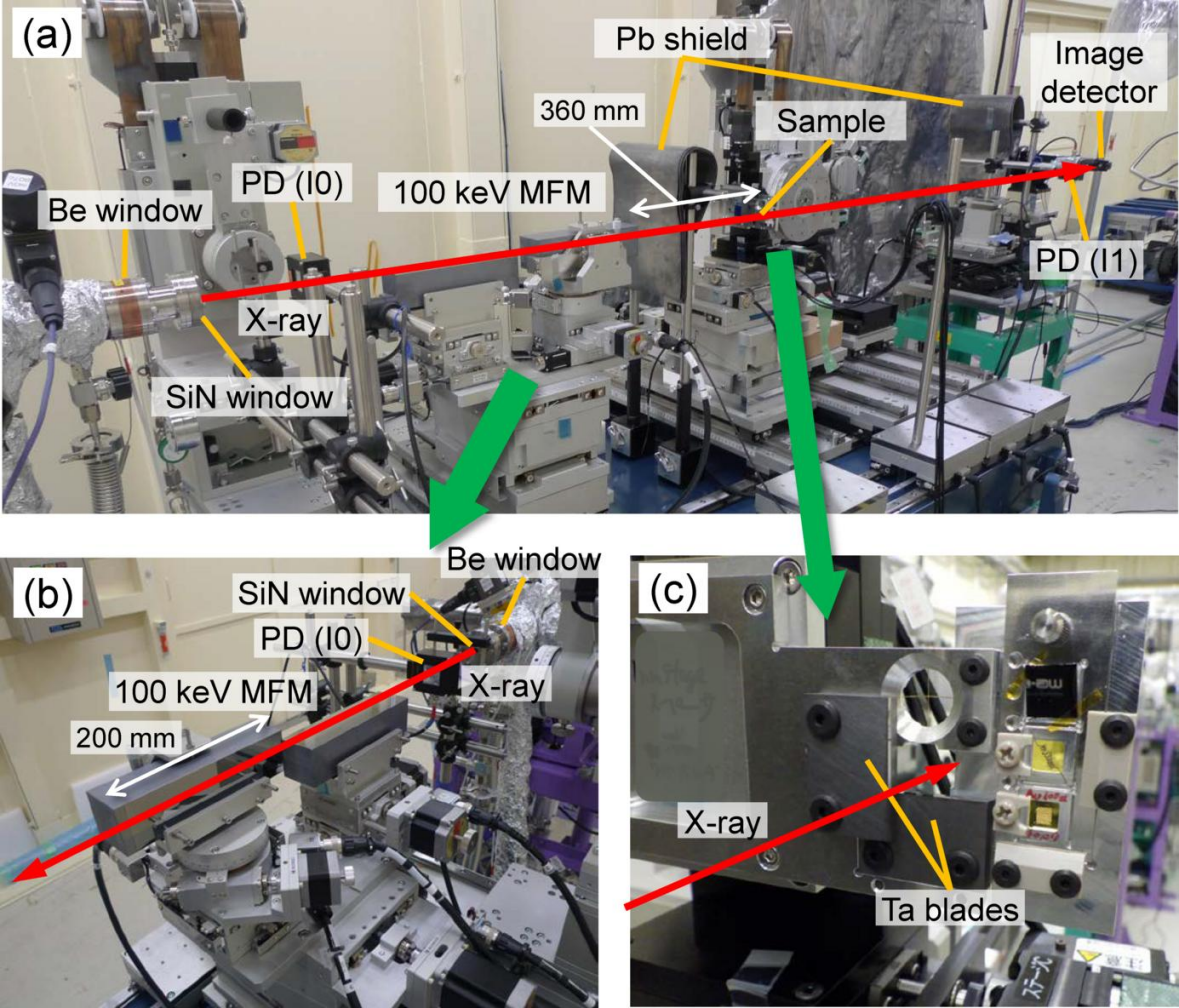
(*a*) Photograph of the area around the multilayer focusing mirrors, the sample stage and the image detector at the R&D area in Fig. 1 ▸. Enlarged photographs of (*b*) the multilayer focusing mirrors and (*c*) the tantalum blades attached on the sample scanning stage.

**Figure 8:**
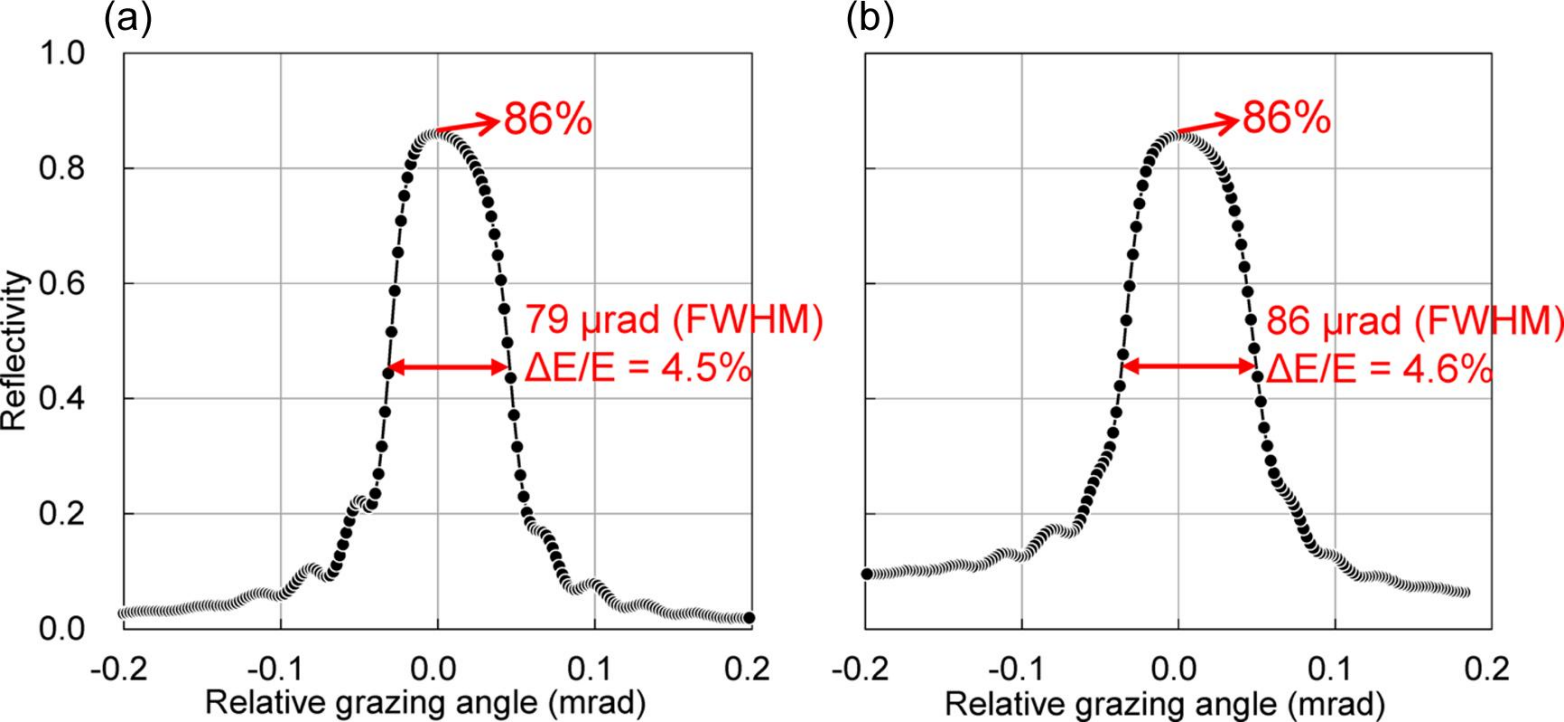
The measured result of reflectivity curves by varying the grazing angle of (*a*) Mv and (*b*) Mh.

The focusing mirrors were installed in OH2 of BL05XU, as shown in Fig. 7 ▸. The mirror environment was atmospheric, without a vacuum or gas chamber. We measured the reflectivity, focusing-beam size and flux. The reflectivity curves were measured as functions of the grazing angles of Mv and Mh, as shown in Fig. 8 ▸. The peak reflectivity of each mirror was 86%, resulting in a maximum value of 74% for the two bounce reflections. The reflection width of Mv (Mh) was 79 (86) µrad in FWHM, which corresponds to an energy bandwidth of Δ*E*/*E* = 4.5% (4.6%). These values were sufficiently wider than the DMM energy bandwidth of 1%.

**Figure 9:**
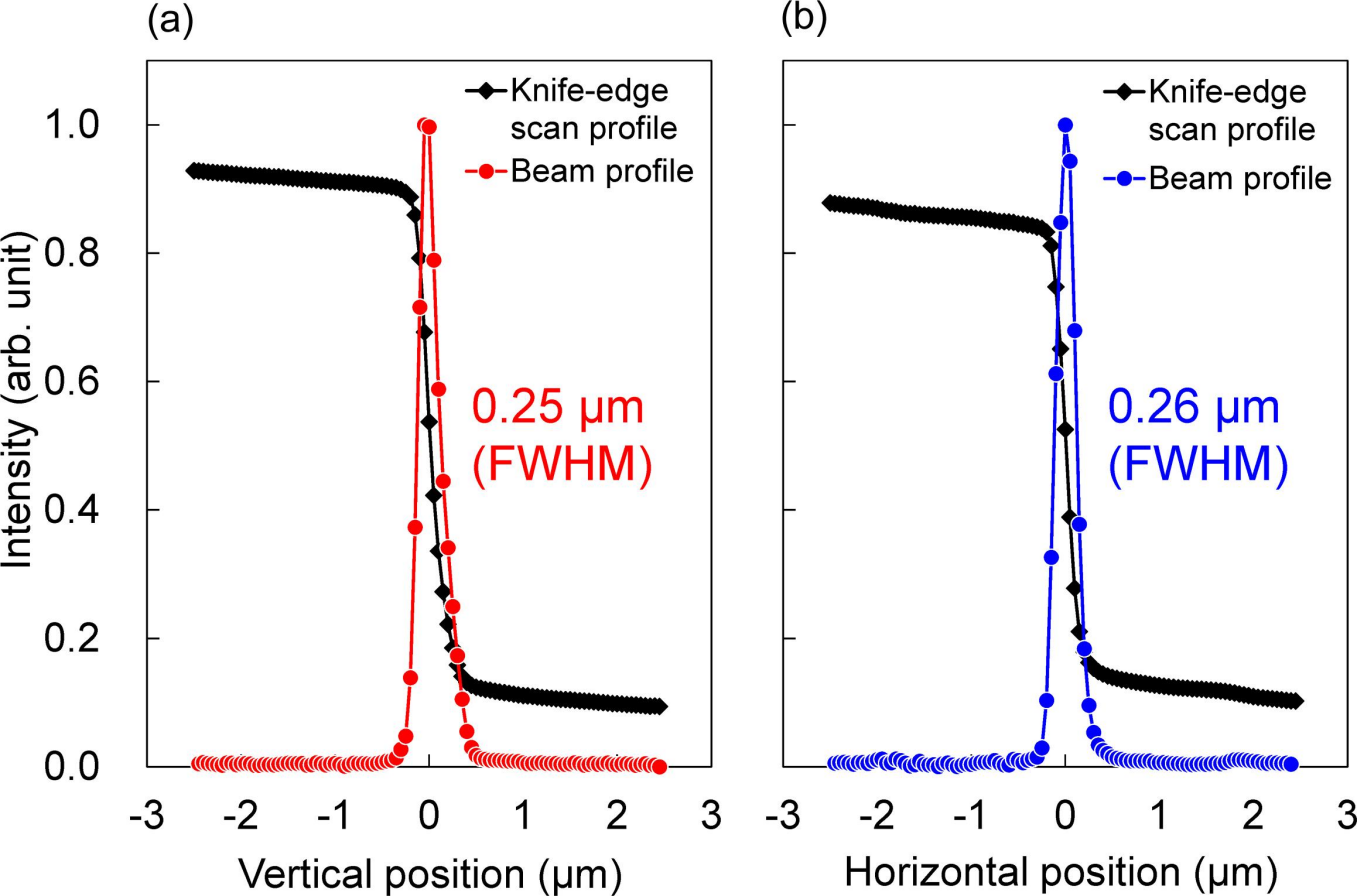
The measured result of focusing-beam profiles in (*a*) the vertical and (*b*) the horizontal direction.

**Figure 10:**
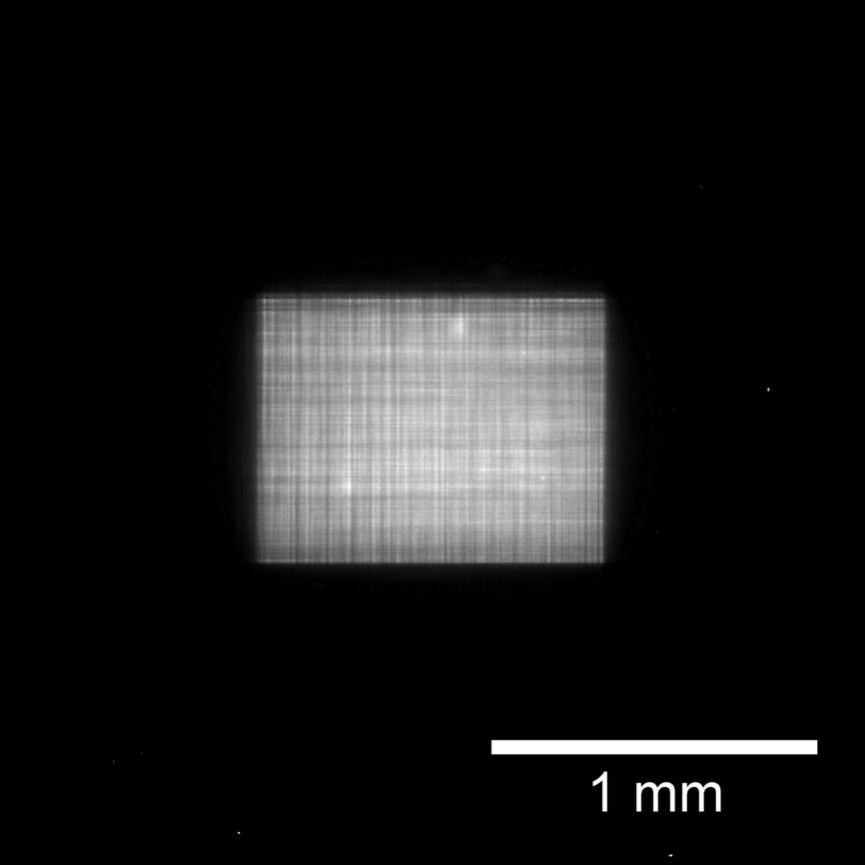
The far-field image of the reflected beam from the focusing mirror. The field of view was 2.66 mm on each side. The aperture size of the FES was 0.35 mm (V) × 0.02 mm (H).

The beam profiles of the focusing beam were measured using a knife-edge scanning method with tantalum blades [shown in Fig. 7 ▸(*c*)]. For the high-spatial-resolution mode with the 20 µm horizontal width of the FES, the beam size was measured to be 0.25 µm (V) × 0.26 µm (H), as shown in Fig. 9 ▸, with a flux of 6 × 10^10^ photons s^−1^. The beam size calculated from the geometric magnification and the blurring due to slope error was 0.25 µm (V) × 0.28 µm (H), which is consistent with the measured size. A far-field image of the beam reflected from the focusing mirror was observed using a high-spatial-resolution imaging detector (Kameshima *et al.*, 2019 ▸) composed of a LuAG:Ce scintillator, an imaging system with ×5 objective lens, and a CMOS camera (ORCA-Flash4.0 v3, Hamamatsu Photonics KK), as shown in Fig. 10 ▸. A clear reflected image without notable ripples was observed, indicating that the figure errors of both the mirror surface and multilayer deposition were sufficiently suppressed.

For the high-flux mode with the horizontal FES opened to be 1.5 mm, the focusing-beam size was measured as 0.32 µm (V) × 5.3 µm (H) with a high flux of 1 × 10^12^ photons s^−1^.

### 3.3. Scanning transmission images

**Figure 11:**
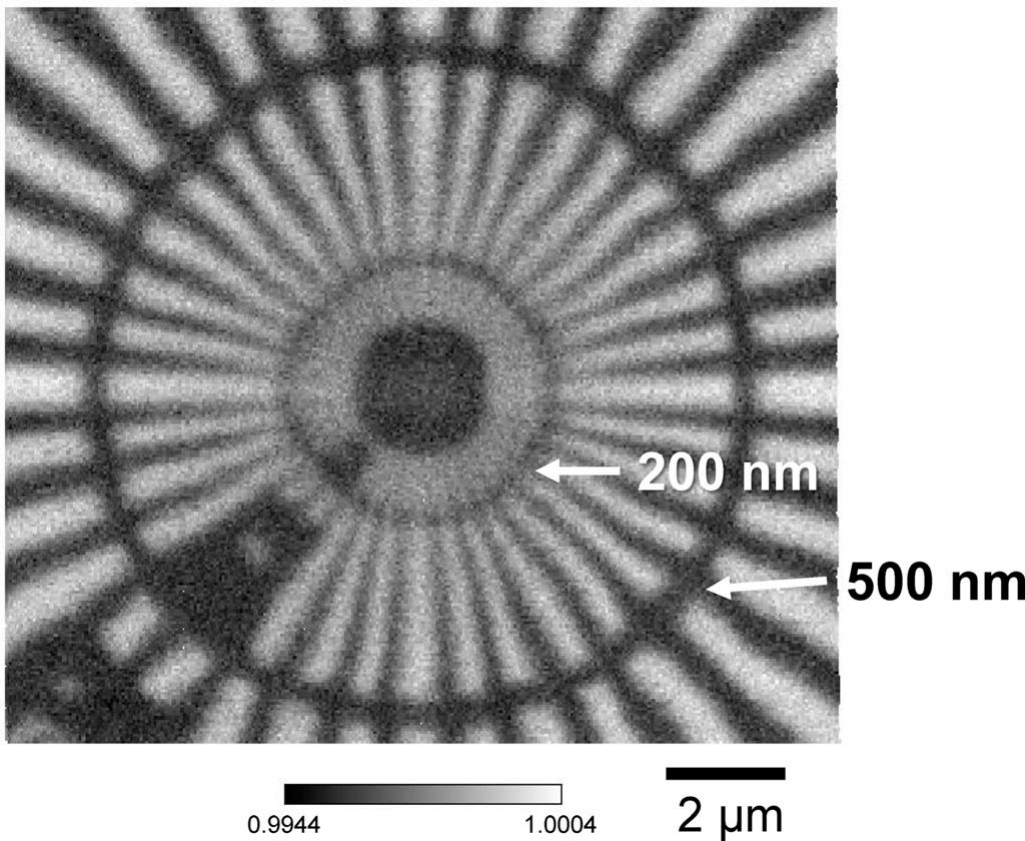
The scanning transmission image of a tantalum Siemens star chart. We used 100 keV X-rays with a focusing-beam size of 0.25 µm (V) × 0.26 µm (H). The structure of 200 nm line and space was clearly observed.

**Figure 12:**
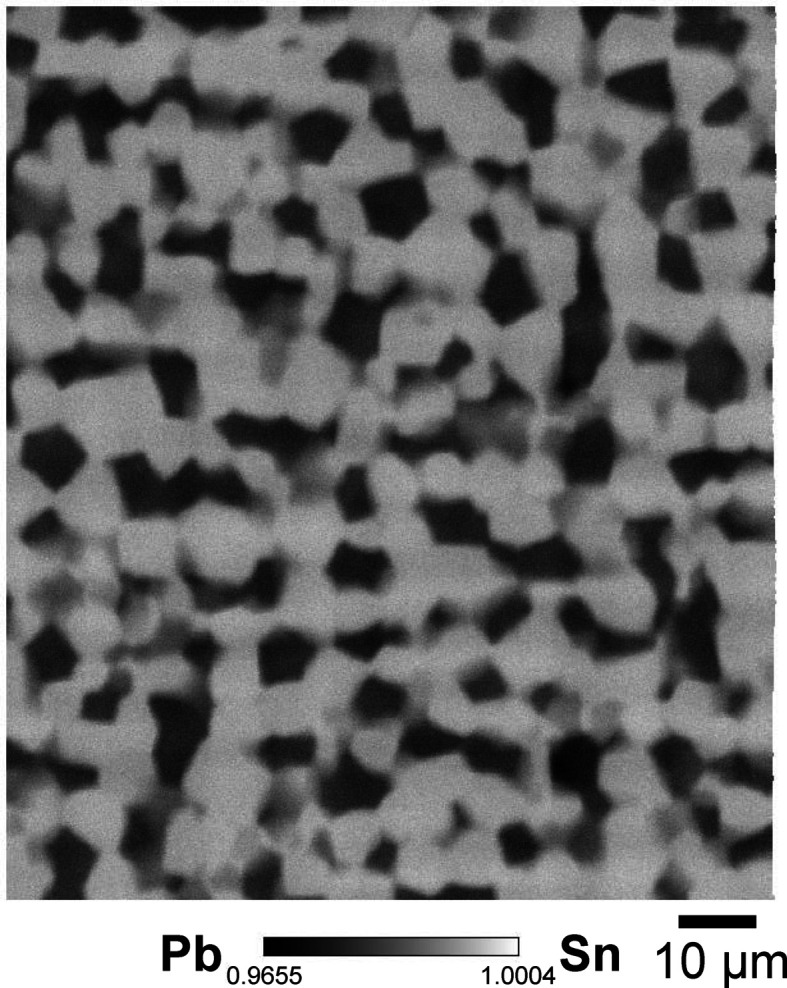
The scanning transmission image of Sn–Pb solder film. We used 100 keV X-rays with a focusing-beam size of 0.25 µm (V) × 0.26 µm (H). Eutectic grain structures, several micrometres in size, consisting of lead and tin, were clearly observed.

As a test, we measured scanning transmission images of a tantalum Siemens star chart (XRESO-100, NTT Advanced Technology Corporation) with a tantalum thickness of 1 µm at the high-spatial-resolution mode, as shown in Fig. 11 ▸. The structure of the 200 nm line and space was resolved without noticeable astigmatism. The transmission of the 1 µm-thick tantalum is 99.3%, and a difference of 0.7% can be clearly observed. As another test sample, we measured Sn–Pb solder film with a thickness of 6 µm, as shown in Fig. 12 ▸. Eutectic grain structures, several micrometres in size, consisting of lead and tin, were clearly observed.

## 4. Summary and perspective

We constructed a multilayer KB focusing system at the undulator beamline BL05XU of SPring-8. The focusing mirror system had a wide bandwidth of 5% and a high peak reflectivity of 74%. Performance was evaluated at BL05XU, which produced an intense 100 keV X-ray beam with a bandwidth of 1%. We confirmed that a small beam size of 0.25 µm (V) × 0.26 µm (H) was achieved with a high flux of 6 × 10^10^ photons s^−1^ for 100 keV X-rays at the high-resolution mode. The fine structures of a tantalum Siemens star chart and Sn–Pb solder film were successfully resolved using this system.

For a fourth-generation synchrotron light source with a smaller source size, a small horizontal focus can be achieved without using a secondary source formed by the FES, which significantly enhances the available flux. Moreover, the undulator spectrum is composed of single peaks without satellite profiles. The multilayer KB focusing system enables the extraction of a specific harmonic with Δ*E*/*E* of a few percent, which further increases the intensity.

## Figures and Tables

**Table 1:** Parameters of the focusing mirrors

	Vertical	Horizontal
Name	Mv	Mh
Substrate material	Si	
Substrate size	200 mm × 50 mm × 50 mm	
Effective area	190 mm × 10 mm	
Surface shape	Elliptical cylinder	
Focal length	680 mm	460 mm
Grazing angle at mirror center	1.85 mrad	1.77 mrad
Acceptance aperture	∼360 µm	∼340 µm
Coating	[W/C]_50_ laterally graded multilayer	
Multilayer period, *d*	3.24–3.8 nm	3.24–4.15 nm
Thickness ratio (W thickness/*d*)	0.5	
Demagnification	1/91	1/135, 1/72†

† When the FES is used as a secondary source.
